# Integrated clinicopathologic and molecular analysis of endometrial carcinoma: Prognostic impact of the new ESGO-ESTRO-ESP endometrial cancer risk classification and proposal of histopathologic algorithm for its implementation in clinical practice

**DOI:** 10.3389/fmed.2023.1146499

**Published:** 2023-03-30

**Authors:** Dario de Biase, Thais Maloberti, Angelo Gianluca Corradini, Francesca Rosini, Marco Grillini, Martina Ruscelli, Sara Coluccelli, Annalisa Altimari, Elisa Gruppioni, Viviana Sanza, Daniela Turchetti, Andrea Galuppi, Martina Ferioli, Susanna Giunchi, Giulia Dondi, Marco Tesei, Gloria Ravegnini, Francesca Abbati, Daniela Rubino, Claudio Zamagni, Pierandrea De Iaco, Donatella Santini, Claudio Ceccarelli, Anna Myriam Perrone, Giovanni Tallini, Antonio De Leo

**Affiliations:** ^1^Solid Tumor Molecular Pathology Laboratory, IRCCS Azienda Ospedaliero-Universitaria di Bologna, Bologna, Italy; ^2^Department of Pharmacy and Biotechnology (FaBit), University of Bologna, Bologna, Italy; ^3^Department of Medical and Surgical Sciences (DIMEC), University of Bologna, Bologna, Italy; ^4^Pathology Unit, IRCCS Azienda Ospedaliero-Universitaria di Bologna, Bologna, Italy; ^5^Unit of Medical Genetics, IRCCS Azienda Ospedaliero-Universitaria di Bologna, Bologna, Italy; ^6^Radiation Oncology, IRCCS Azienda Ospedaliero-Universitaria di Bologna, Bologna, Italy; ^7^Division of Gynecologic Oncology, IRCCS Azienda Ospedaliero-Universitaria di Bologna, Bologna, Italy; ^8^IRCCS Azienda Ospedaliero-Universitaria di Bologna, Bologna, Italy

**Keywords:** endometrial carcinoma, molecular classification, prognosis, risk stratification, histopathologic parameters

## Abstract

**Introduction:**

The European Society of Gynecologic Oncology/European Society of Radiation Therapy and Oncology/European Society of Pathology (ESGO/ESTRO/ESP) committee recently proposed a new risk stratification system for endometrial carcinoma (EC) patients that incorporates clinicopathologic and molecular features. The aim of the study is to compare the new ESGO/ESTRO/ESP risk classification system with the previous 2016 recommendations, evaluating the impact of molecular classification and defining a new algorithm for selecting cases for molecular analysis to assign the appropriate risk class.

**Methods:**

The cohort included 211 consecutive EC patients. Immunohistochemistry and next-generation sequencing were used to assign molecular subgroups of EC: *POLE* mutant (*POLE*), mismatch repair deficient (MMRd), p53 mutant (p53abn), and no specific molecular profile (NSMP).

**Results:**

Immuno-molecular analysis was successful in all cases, identifying the four molecular subgroups: 7.6% *POLE*, 32.2% MMRd, 20.9% p53abn, and 39.3% NSMP. The recent 2020 guidelines showed a 32.7% risk group change compared with the previous 2016 classification system: the reassignment is due to *POLE* mutations, abnormal p53 expression, and a better definition of lymphovascular space invasion. The 2020 system assigns more patients to lower-risk groups (42.2%) than the 2016 recommendation (25.6%). Considering the 2020 risk classification system that includes the difference between “unknown molecular classification” and “known,” the integration of molecular subgroups allowed 6.6% of patients to be recategorized into a different risk class. In addition, the use of the proposed algorithm based on histopathologic parameters would have resulted in a 62.6% reduction in molecular analysis, compared to applying molecular classification to all patients.

**Conclusion:**

Application of the new 2020 risk classification integrating clinicopathologic and molecular parameters provided more accurate identification of low-and high-risk patients, potentially allowing a more specific selection of patients for post-operative adjuvant therapy. The proposed histopathologic algorithm significantly decreases the number of tests needed and could be a promising tool for cost reduction without compromising prognostic stratification.

## 1. Introduction

Endometrial carcinoma (EC) represents the most common gynecologic cancer in Western countries, with a frequency of 15 to 25 per 100,000 women (1, 2). In the majority of cases, patients present at diagnosis with an early-stage tumor and excellent prognosis. However, 15–20% of cases may have high-risk disease recurrence with an aggressive clinical course. Prognostic stratification is conventionally based on clinicopathologic parameters (e.g., histotype, grade, stage) also used to define the therapeutic approach. In recent years, the molecular understanding of EC has undergone impressive development. The Cancer Genome Atlas (TCGA) endometrial collaborative project identified four distinct prognostic EC groups based on molecular alterations: (i) the “ultramutated” subtype, characterized by *POLE* exonuclease domain mutation (*POLE*) with excellent prognosis; (ii) the “hypermutated” subtype, defined by MisMatch Repair deficiency (MMRd) with intermediate prognosis; (iii) the “copy-number high” subtype, with p53 abnormal expression (p53abn) and poor prognosis; (iv) the “copy-number low subtype,” also known as No Specific Molecular Profile-NSMP with intermediate prognosis (3). Two groups (ProMisE and PORTEC) have proposed and validated molecular classification tools based on surrogate markers (*POLE* mutation, microsatellite instability, and p53 alteration) that can identify the four molecular classes similar to those reported in the TCGA study (4–6). In 2020, the European Society of Gynecological Oncology (ESGO), the European Society for Radiotherapy and Oncology (ESTRO), and the European Society of Pathology (ESP) published revised guidelines for risk group assessment in endometrial cancer, integrating both molecular markers and clinicopathologic parameters in order to improve and personalize patient treatment (7). These molecular prognostic risk groups represent a revolutionary milestone in the management of patients with EC and will require a radical change in the diagnostic and therapeutic approaches to this cancer. Previously, the 2016 recommendations proposed a prognostic stratification system based exclusively on conventional clinicopathologic parameters such as tumor histotype, stage of disease, grade, and lymphovascular space invasion (8). The new 2020 guidelines represent an integrated clinical and molecular system for the prognostic definition of endometrial carcinoma. The new risk assessment serves as a basis for patient management, in particular for a more appropriate definition of adjuvant therapy. However, the implementation of risk groups involves the introduction of molecular tests into clinical practice, the impact of which can lead to a sometimes surprising change in risk classes, and which must be correctly interpreted by the care team.

The objectives of the present study include (I) the evaluation of the prognostic impact of the new ESGO/ESTRO/ESP 2020 guidelines incorporating molecular classification in a consecutive cohort of EC patients; (II) a comparison of the new risk groups with the non-molecular, clinicopathologic-only risk groups of 2016; (III) definition of a new algorithm for selection of cases to be submitted for molecular analysis for assignment of the correct risk class and, consequently, indication for appropriate adjuvant treatment.

## 2. Materials and methods

### 2.1. Cohort and clinicopathologic data

We retrospectively analyzed data from the cohort of patients surgically treated at the Division of Gynecologic Oncology of “IRCCS Azienda Ospedaliero-Universitaria di Bologna” (Bologna, Italy) (9). A subset of this cohort was studied in a preliminary study on endometrial carcinoma by our group (10). The local ethics committee CE-AVEC (Comitato Etico-Area Vasta Emilia Centro) approved the present study (registration n. 27/2019/Sper/AOUBo). All patients provided their written agreement to use of their tissues and data for the study. A total of 211 consecutive cases of primary endometrial carcinoma were included in the study, and for each, a representative Formalin-fixed paraffin-embedded (FFPE) tissue block was retrieved from the files of the Pathology Unit of “IRCCS Azienda Ospedaliero-Universitaria di Bologna” (Bologna, Italy). Two expert pathologists (D.S., A.D.L.) thoroughly reviewed and examined histology slides and all histopathologic parameters. Clinicopathologic findings including age at diagnosis, Body Mass Index (BMI), International Federation of Gynecology and Obstetrics (FIGO) stage, and follow-up data were obtained from clinical, surgical, and pathologic records reported in a comprehensive clinicopathologic database. Following the classification of tumors by the World Health Organization, ECs were categorized using standard histopathologic criteria (11–13), graded, and staged using standard FIGO criteria (14, 15). Lymphovascular space invasion (LVSI) was defined by the presence of tumor cells within endothelial-lined vascular/lymphatic spaces outside the tumor invasive border. Two tiers of semi-quantitative scoring were used: no LVSI/focal (a single focus of LVSI recognized around the tumor), and substantial (diffuse or multifocal LVSI around the tumor) (16, 17).

### 2.2. Immunohistochemistry

Immunohistochemical (IHC) analysis included assessment of the following markers: p53, PTEN, MLH1, PMS2, MSH2, MSH6, and Ki67. Details of the IHC antibodies are described in the Supplementary material.

Immunohistochemical staining of p53 was classified as normal (wild-type) or abnormal/mutant-like (p53abn). A case was classified as p53abn if one of the following aberrant patterns was observed: (i) protein overexpression, (ii) “null” phenotype, or (iii) positive cytoplasmic staining (18–20).

PTEN cases were defined: (i) positive, if uniform or heterogeneous staining was found in the neoplastic cells; (ii) negative if no cytoplasmic/nuclear immunostaining was found in the neoplastic cells (21). The mismatch repair proteins (MLH1, PMS2, MSH2, and MSH6) were scored negative if no nuclear immunostaining was present. Cases were considered mismatch repair deficient (MMRd) if one of the four proteins was absent, or if the staining for MLH1/PMS2 or MSH2/MSH6 were negative (22).

The evaluation of the proliferative index (Ki67) in neoplastic cells was carried out quantitatively using image analysis with the Image-Pro Plus 5.1 software (Media Cybernetics Inc., Silver Spring, MD, USA). The analysis was performed in at least 40 ×200 magnification fields, and the Ki67 score was expressed as ratio (%) between positive neoplastic cells and total neoplastic cells (23).

### 2.3. DNA extraction and next-generation sequencing

DNA was extracted starting from two to four 10-μm-thick FFPE tissue sections, according to the areas of interest marked on the control hematoxylin and eosin (H&E) stained slide. DNA was extracted using the Quick Extract Kit (Epicentre, Madison, WI, United States) and quantified by “Qubit” fluorometer (ThermoFisher Scientific, Waltham, MA, United States). About 30 ng of gDNA was amplified using a laboratory-developed panel, including the following genomic regions (human reference sequence hg19/GRCh37, total of 169 amplicons, 12.74 kb): *ARID1A* (all CDS region), *BRAF* (exon 15), *cKIT* (exons 8, 9, 11, 13, 17), *CTNNB1* (exons 3, 7, 8), *HRAS* (exons 2–4), *KRAS* (exons 2–4), *NRAS* (exons 2–4), *PIK3CA* (exons 10, 21), *POLE* (exons 9–14), and *TP53* (exons 4–9) (23). Template preparation was performed using the Chef Machine instrument (ThermoFisher Scientific) and then sequenced using an Ion 530 chip run with a Gene Studio S5 Prime sequencer (ThermoFisher Scientific), according to the manufacturer’s instruction (ThermoFisher Scientific), as previously described (10, 24). Only nucleotide variations detected in at least 5% of the total number of reads analyzed, and observed in both strands, were considered for the mutational call. The sequences obtained were analyzed using the Ion Reporter Software (version 5.18, ThermoFisher Scientific) and the Integrative Genomics Viewer 2.12.2 (IGV) tool (Available online:^1^–accessed on January 2023). The pathogenicity of each mutation was checked using the Varsome database (^2^accessed on January 2023).

### 2.4. Molecular classification

**Figure 1:**
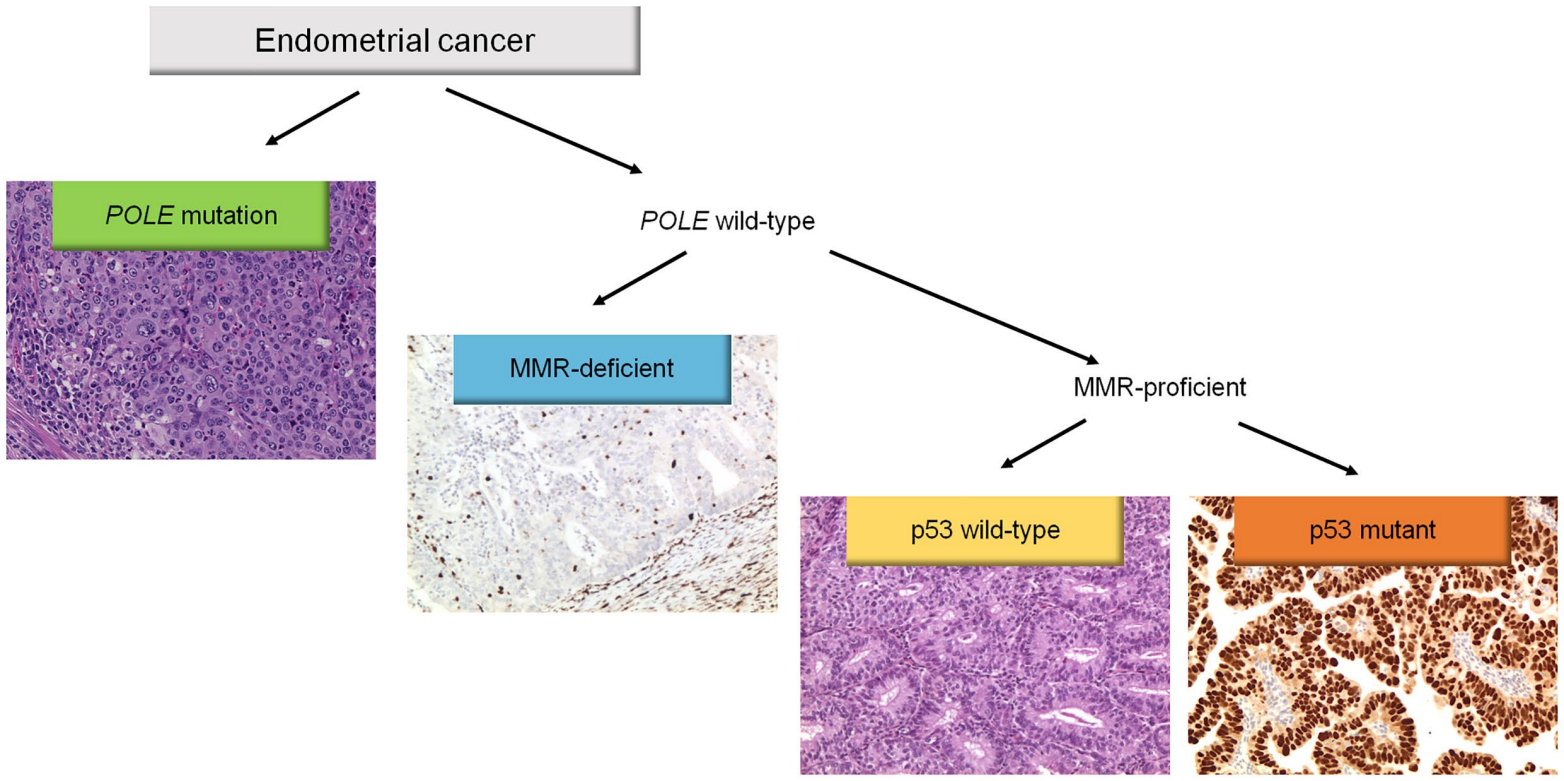
Diagnostic algorithm for the integrated histomolecular endometrial carcinoma classification. This algorithm can be applied for all histological endometrial cancer histotypes (including carcinosarcoma).

Molecular classification was applied following the WHO Classification of Female Genital Tumors (13, 25, 26) (see Figure 1). Cases were classified as: (i) *POLE*, (ii) MMRd, (iii) p53abn, (iv) NSMP. First, all cases were tested for *POLE* mutations. The diagnostic interpretation of *POLE* mutations was based according to reported guidelines (27). The *POLE* analysis allowed identifying the “ultramutated” group tumors (*POLE*). Then, immunohistochemical analysis for MMR proteins was performed to identify MMR deficient (MMRd) tumors and to assign these tumors to “hypermutated” group (in absence of *POLE* mutations). Subsequently, IHC for p53 was evaluated to detect p53abn tumors. These p53abn tumors correspond to the “copy-number high/serous-like” molecular subgroup. “No specific molecular profile” (NSMP) tumors were those exhibiting normal p53 and MMR expression by IHC and with no *POLE* mutations and corresponded to the “copy-number low” subgroup.

### 2.5. Prognostic risk grouping

All cases were categorized according to the previous ESMO 2016 recommendations and to the novel 2020 ESGO/ESTRO/ESP guidelines in one of five risk groups with and without the integration of the molecular classification (see supplementary Table 1) (7, 8).

### 2.6. Statistics

Summary statistics are reported as numbers (percentages) or mean ± standard deviation (SD). *χ*^2^ test, t-test, Fisher’s exact test, Kruskal–Wallis test, and Mann–Whitney test were applied for comparisons between groups. Survival curves were calculated using the Kaplan–Meier method with log-rank test: all recurrences (local, regional, and distant) were considered as an event. All reported *p* values were based on two-sided tests with *p* < 0.05 considered statistically significant. Analyses were performed using Stata software, version 15 (Stata Statistical Software: Release 15, 2017; StataCorp LLP, College Station, TX, United States).

## 3. Results

### 3.1. Conventional clinicopathologic parameters of endometrial carcinoma cohort

The clinicopathologic characteristics of the 211 patients are shown in Table 1. The median patient age at diagnosis was 62.4 years (range 34–86). The median body mass index (BMI; kg/m2) was 28.3 (18.3–55.2). Histologic classification includes: 161 (76.3%) endometrioid carcinomas, 25 (11.8%) dedifferentiated/undifferentiated, 19 (9%) serous, 3 (1.4%) clear cell carcinomas, and 3 (1.4%) carcinosarcomas. Grade distribution includes: 125 (58.3%) low-grade (FIGO grade 1 and 2), and 88 (41.7%) high-grade (FIGO grade 3) tumors. Lymph node metastases were detected in 33 (15.6%) patients.

**Table 1:** Clinicopathologic characteristics of the study sample.

Clinicopathologic characteristics	*n* = 211 (%)
Age, years	62.4 ± 10.4
Body mass index, kg/m^2^	28.3 ± 7.3
**Tumor type**	
Endometrioid	161 (76.3)
Dedifferentiated/Undifferentiated	25 (11.8)
Serous	19 (9.0)
Carcinosarcoma	3 (1.4)
Clear cell	3 (1.4)
**Grade**	
Low	123 (58.3)
High	88 (41.7)
**Depth of invasion**	
<50%	146 (69.1)
≥50%	65 (30.8)
**Lymphovascular space invasion (LVSI)**	
Absent	70 (33.2)
Focal	66 (31.3)
Substantial	75 (35.5)
**Lymph node status**	
Negative	172 (81.5)
Positive	33 (15.6)
Unknown/Not tested	6 (2.8)
**FIGO stage**	
IA	119 (56.4)
IB	32 (15.2)
II	10 (4.7)
III	42 (19.9)
IV	8 (3.8)
**Recurrence**	
Absent	184 (87.2)
Present	27 (12.8)
**Survival**	
Alive	203 (96.2)
DOD	8 (4.3)
**ESMO 2016**	
Low risk	52 (24.6)
Intermediate risk	2 (0.9)
High-intermediate risk	70 (33.2)
High risk	80 (37.9)
Advanced/metastatic disease	7 (3.3)
**ESGO/ESTRO/ESP 2020 molecular classification unknown**	
Low risk	82 (38.8)
Intermediate risk	24 (11.4)
High-intermediate risk	28 (13.3)
High risk	70 (33.2)
Advanced/metastatic disease	7 (3.3)
**ESGO/ESTRO/ESP 2020 molecular classification known**	
Low risk	89 (42.2)
Intermediate risk	18 (8.5)
High-intermediate risk	21 (10.0)
High risk	76 (36.0)
Advanced/metastatic disease	7 (3.3)

Values are counts (percentages) or mean + standard deviation [interquartile range].

DOD, Dead of Disease; LVSI, lymphovascular space invasion.

### 3.2. Application of molecular markers

The molecular classification was feasible in all cases. The application of the immuno-molecular algorithm allowed the identification of four molecular subgroups: *POLE* (*n* = 16; 7.6%), MMRd (*n* = 68; 32.2%), p53abn (*n* = 44; 20.9%), and NSMP (*n* = 83; 39.3%). Of note, 16 cases (7.6%) were categorized as “multiple classifiers.” Specifically, one was *POLE*-mutated and MMRd, 4 were *POLE*-mutated and p53 abnormal, 9 were MMRd and p53-abnormal, and 2 were triple positive (*POLE*-mutated, MMRd, and p53-abnormal). The association of molecular subgroups with clinicopathologic characteristics is summarized in Table 2. As shown, molecular subgroups differ significantly in their clinicopathologic features. Sequencing data are available in NCBI—Sequence Read Archive (SRA) (PRJNA932605) (28).

**Table 2:** Clinicopathologic characteristics of molecular subgroups.

Clinicopathologic characteristics	*POLE*	MMRd	p53abn	NSMP	*p*-value
	(*n* = 16; 7.6%)	(*n* = 68; 32.2%)	(*n* = 44; 20.9%)	(*n* = 83; 39.3%)	
Age, years	58.6 ± 11.6	63.0 ± 9.4	66.9 ± 10.3	60.2 ± 10.2	0.0025
Body mass index, kg/m^2^	27.2 ± 9.3	28.0 ± 7.3	25.5 ± 4.4	30.3 ± 8.1	0.0083
Tumor type					<0.0001
Endometrioid	13 (81.3)	54 (79.4)	17 (38.6)	77 (92.8)	
Dedifferentiated/ Undifferentiated	3 (18.8)	14 (20.6)	2 (4.5)	6 (7.2)	
Serous	0 (0.0)	0 (0.0)	19 (43.2)	0 (0.0)	
Carcinosarcoma	0 (0.0)	0 (0.0)	3 (6.8)	0 (0.0)	
Clear cell	0 (0.0)	0 (0.0)	3 (6.8)	0 (0.0)	
Grade					<0.001
Low	7 (43.8)	43 (63.2)	1 (2.2)	72 (86.7)	
High	9 (56.3)	25 (36.7)	43 (97.7)	11 (13.3)	
Depth of invasion ≥50%	3 (18.8)	24 (35.3)	19 (43.2)	19 (22.9)	0.06
LVSI	5 (31.3)	27 (39.7)	26 (59.0)	17 (20.5)	0.0002
Lymph node status					0.0029
Negative	15 (93.8)	54 (79.4)	29 (65.9)	74 (89.2)	
Positive	1 (6.3)	12 (17.6)	14 (31.8)	6 (7.2)	
FIGO stage					<0.0001
IA	9 (56.3)	36 (52.9)	14 (31.8)	60 (72.3)	
IB/II	5 (31.3)	16 (23.5)	6 (13.6)	15 (18.1)	
III	2 (12.5)	13 (19.1)	19 (43.2)	8 (9.6)	
IV	0 (0.0)	3 (4.4)	5 (11.4)	0 (0.0)	

Values are counts (percentages) or mean + − standard deviation [interquartile range].

POLE, POLE mutant; MMRd, mismatch repair deficient; p53abn, p53 mutant; NSMP, no specific molecular profile; LVSI, lymphovascular space invasion.

### 3.3. Comparison of ESGO/ESTRO/ESP 2020 and ESMO 2016 risk classification systems

Application of the ESGO/ESTRO/ESP 2020 guidelines incorporating molecular classification resulted in the following distribution in the five prognostic risk groups: low-risk *N* = 89 (42.2%), intermediate-risk *N* = 18 (8.5%), high-intermediate *N* = 21 (9.9%), high-risk *N* = 76 (36%) and advanced *N* = 7 (3.3%). A detailed comparison of the 2020 guidelines (with and without molecular classification) and the 2016 clinicopathologic-only risk classification system is shown in Table 3. The ESGO/ESTRO/ESP 2020 guidelines with molecular subgroups resulted in a migration of 69 (32.7%) patients compared to the previous 2016 risk system. By considering the new guidelines, the addition of molecular classification resulted in a change of risk class in 14 (6.6%) patients compared to risk categorization based on histopathologic parameters alone.

**Table 3:** Number of patients classified into risk groups according to ESGO/ESTRO/ESP 2020 guidelines and to 2016 recommendations.

	ESGO/ESTRO/ESP 2020 molecular classification known					
	Low risk	Intermediate risk	High-intermediate risk	High risk	Advanced/ metastatic	Total
**ESMO 2016**						
Low risk	52	0	0	0	0	52
Intermediate risk	0	2	0	0	0	2
High-intermediate risk	32	17	14	8	0	70
High risk	5	0	7	68	0	80
Advanced/metastatic	0	0	0	0	7	7
**ESGO/ESTRO/ESP 2020 molecular classification unknown**						
Low risk	82	0	0	0	0	82
Intermediate risk	1	18	0	5	0	24
High-intermediate risk	5	0	21	2	0	28
High risk	1	0	0	69	0	70
Advanced/metastatic	0	0	0	0	7	7
Total	89	18	21	76	7	211

#### 3.3.1. ESGO/ESTRO/ESP 2020 molecular classification known versus ESMO 2016 recommendations

**Figure 2:**
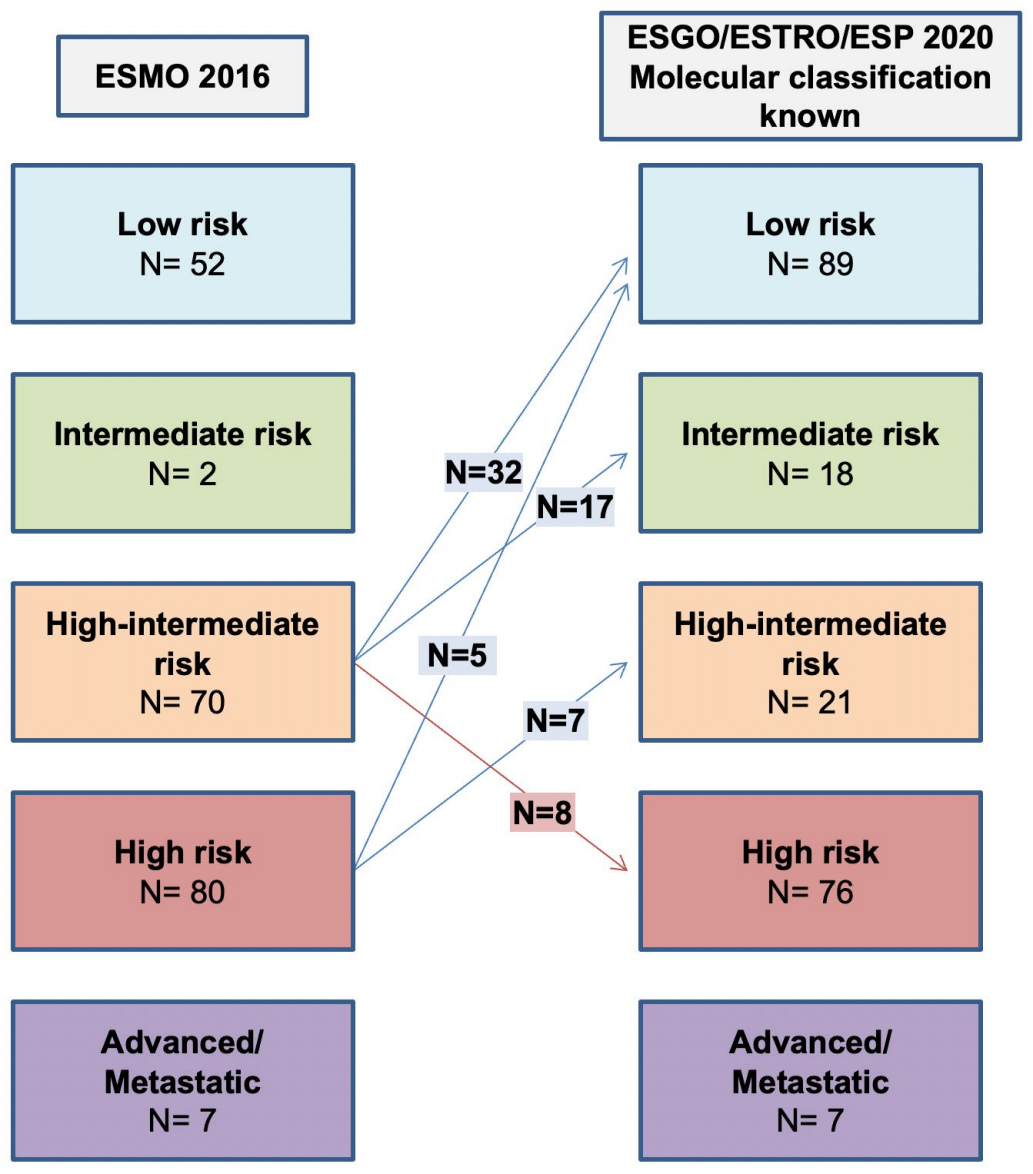
Comparison between ESMO 2016 recommendations and ESGO/ESTRO/ESP 2020 guidelines with molecular classification known.

As shown in Table 3 and Figure 2, the ESGO/ESTRO/ESP 2020 guidelines with molecular subgroups result in reallocation of 61 (28.9%) patients to a lower-risk group, while 8 (3.8%) are assigned to a higher-risk group compared with the 2016 ESMO recommendations. Overall, 17/69 (24.6%) cases were reclassified due to molecular subgroups, 45/69 (65.2%) cases were reallocated into a different risk class due to re-evaluation of histopathologic parameters (LVSI and stage), and 7/69 (10.2%) cases were categorized in a different risk group for both (molecular and histopathologic parameters). Specifically: 32 cases previously classified as “high-intermediate risk” according to ESMO 2016 are reclassified to “low risk” following the 2020 guidelines, in 4 cases due to the presence of *POLE* mutation and in 28 cases due to a redefinition of lymphovascular invasion as focal; 17 “high-intermediate risk” cases according to ESMO 2016 are classified as “intermediate risk” according to the 2020 guidelines due to a redefinition of lymphovascular invasion as focal; 8 “high-intermediate risk” cases according to ESMO 2016 are reallocated to “high risk” because they fall into the p53abn molecular subgroup; 5 “high risk” cases according to ESMO 2016 are reclassified to “low risk” due to the presence of POLE mutation; 7 “high risk” cases according to ESMO 2016 are defined as “intermediate risk” according to 2020 guidelines (3 MMRd and 4 NSMP stage II).

#### 3.3.2. ESGO/ESTRO/ESP 2020 molecular classification known versus ESGO/ESTRO/ESP 2020 molecular classification unknown

**Figure 3:**
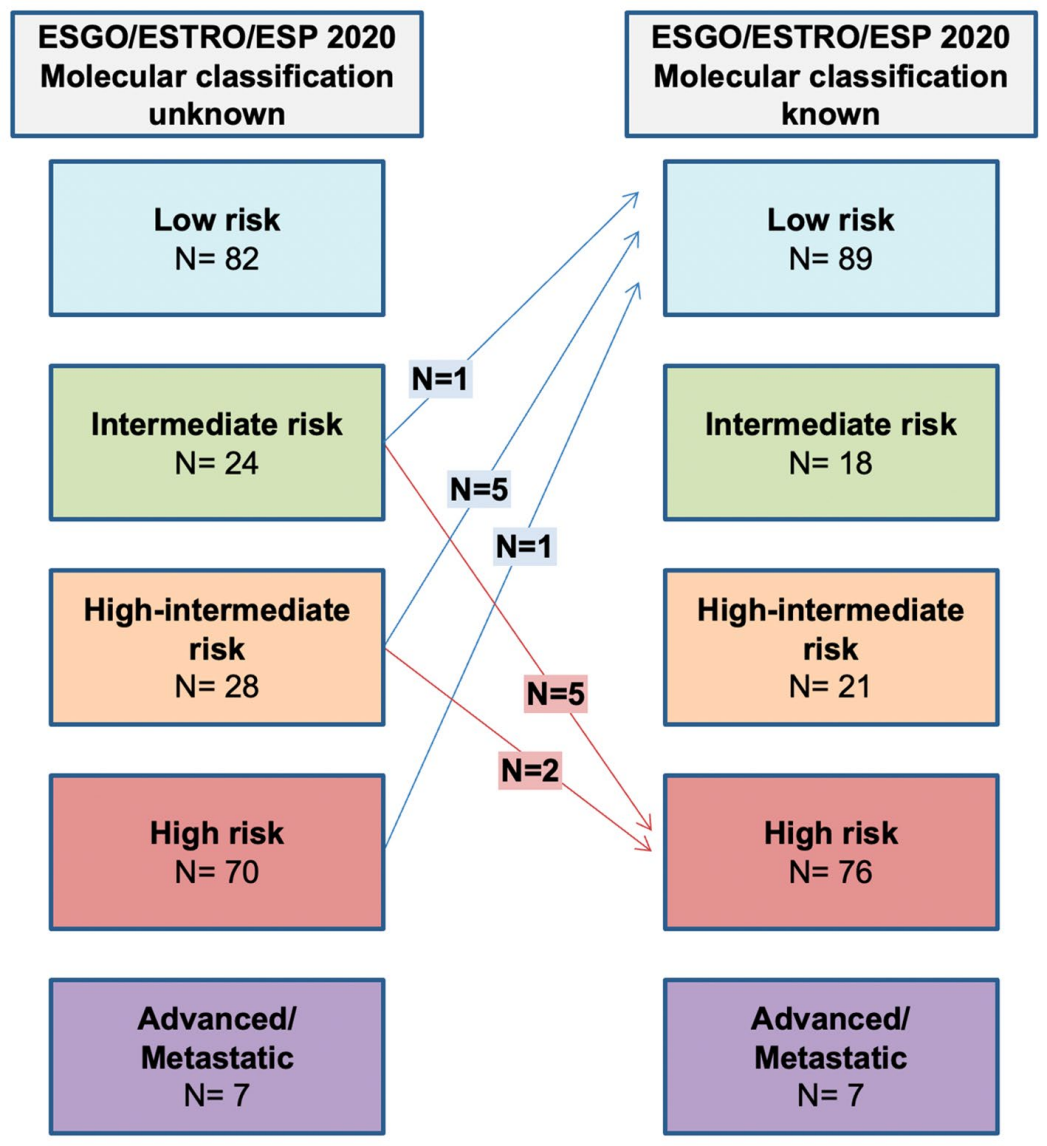
Comparison between ESGO/ESTRO/ESP risk class with molecular classification unknown and ESGO/ESTRO/ESP risk class with molecular classification known.

Molecular subgroup integration allows 7 (3.3%) patients to be assigned to a lower risk group due to *POLE* mutation, while 7 (3.3%) are assigned to a higher risk group because of p53abn. The shift in class due to molecular classification is shown in Figure 3.

#### 3.3.3. Assessment of prognosis

**Figure 4:**
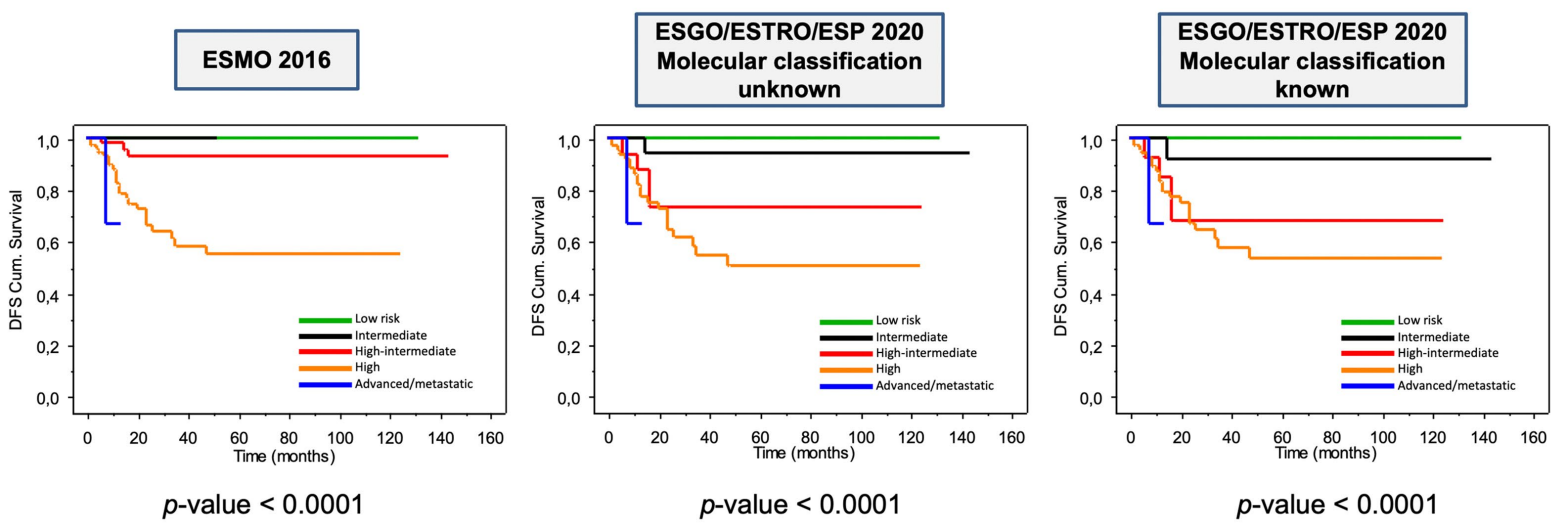
Kaplan–Meier estimations for disease-free survival according to ESMO 2016 and to ESGO/ESTRO/ESP 2020 guidelines.

The prognostic impact of ESGO/ESTRO/ESP 2020 and ESMO 2016 risk classification systems is shown in Figure 4. As can be noted, all risk classification systems show a significant difference among the groups when considering disease-free survival (DFS) (all log-rank *p* < 0.0001). Overall survival was not considered because of the relatively short follow-up time (median DFS ± St.Dev. 22.0 ± 29.9 months) and few disease-related deaths (8–3.8%). Nevertheless, it is evident how the new guidelines provide better prognostic discrimination of each risk class.

### 3.4. Definition of a new algorithm for the selection of cases to be submitted for molecular analysis

**Figure 5:**
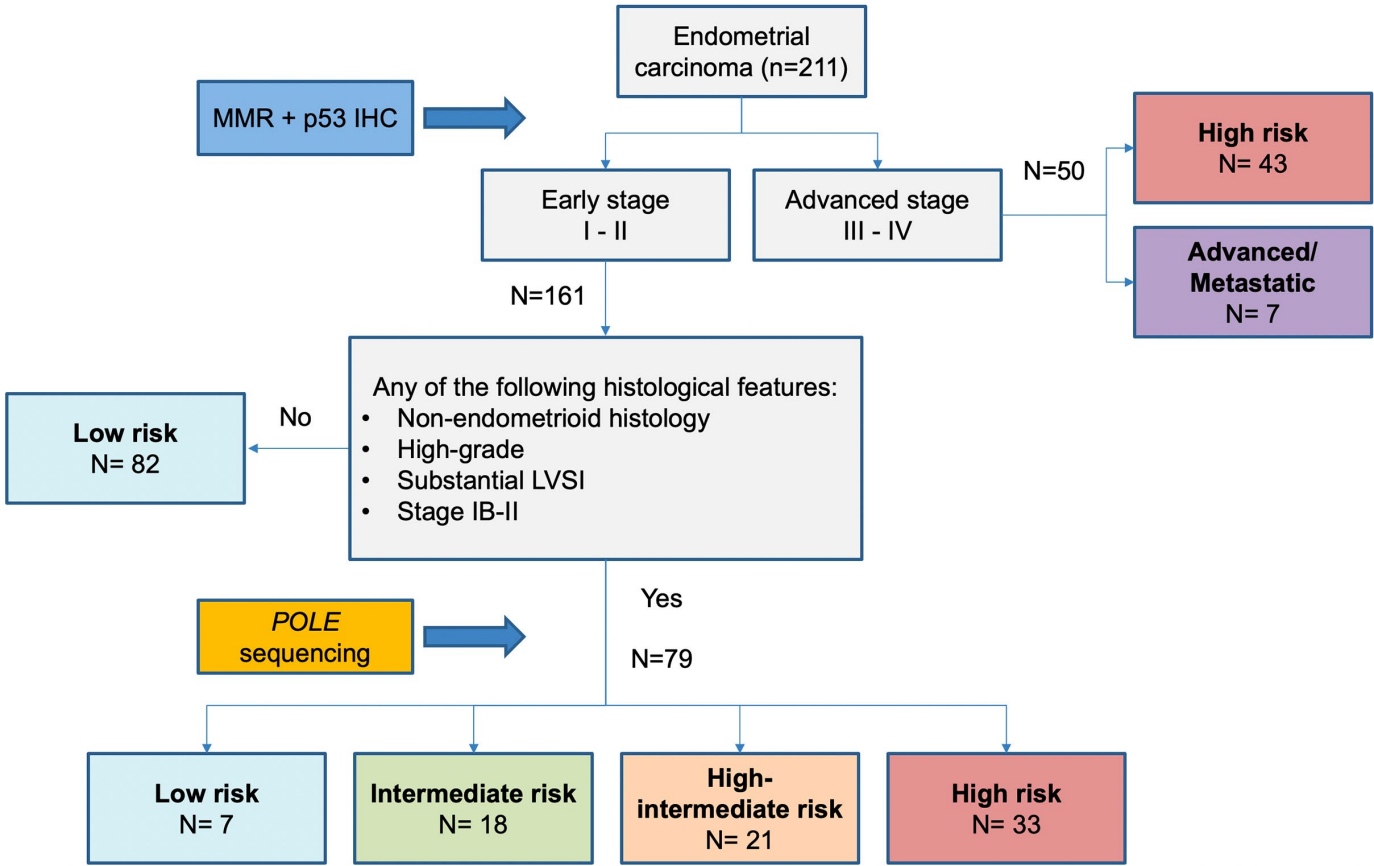
Proposed algorithm based on histopathologic parameters to select only those cases that need molecular analysis for the proper definition of risk.

**Figure 6:**
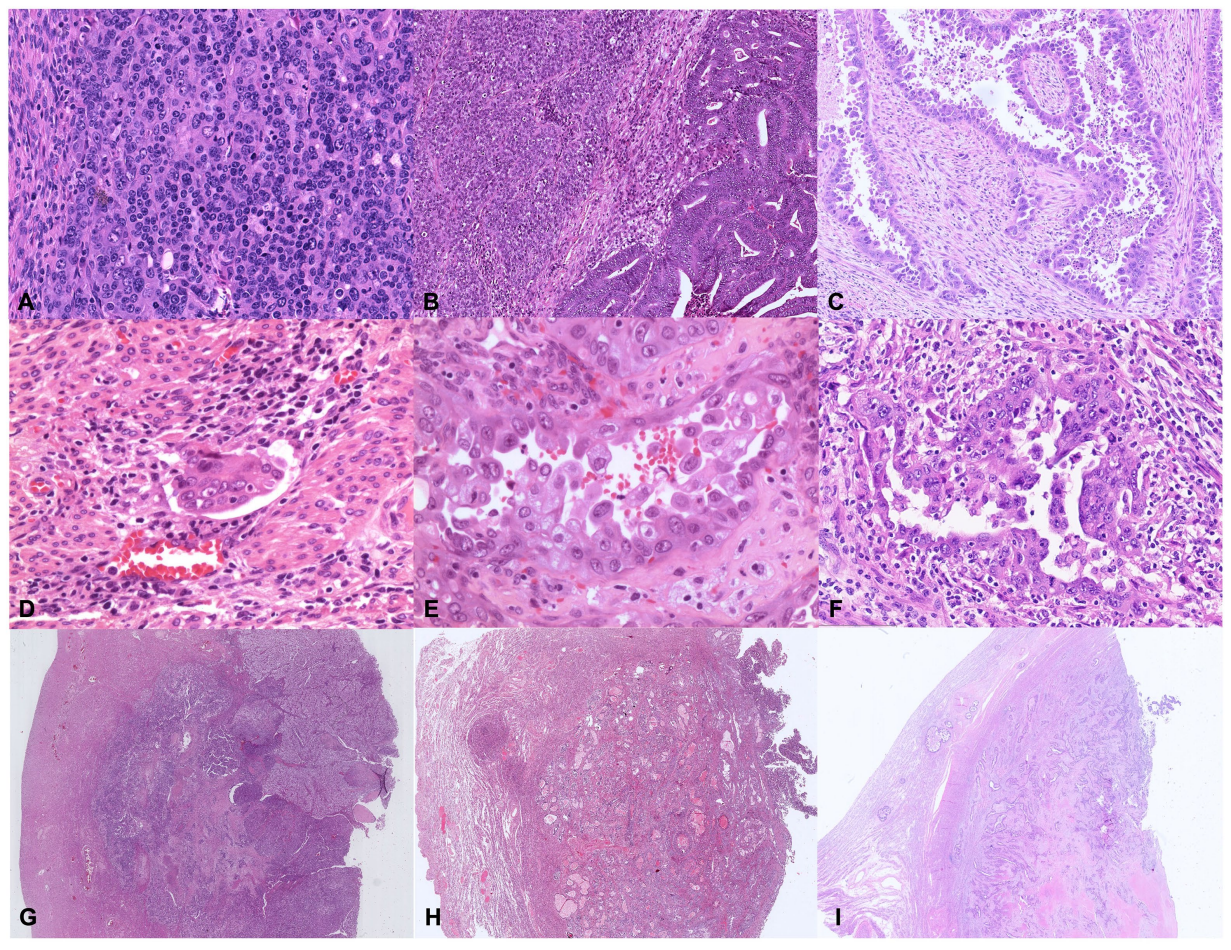
Illustrative histologic pictures of the four histopathologic parameters of the proposed algorithm of different molecular subgroups: **(A)** Undifferentiated *POLE*-mutant EC (Hematoxylin and Eosin; x100 magnification); **(B)** Dedifferentiated MMRd EC (Hematoxylin and Eosin; x100 magnification); **(C)** p53abn serous EC (Hematoxylin and Eosin; x100 magnification); **(D)** Endometrioid NSMP EC with substantial lymphovascular space invasion (Hematoxylin and Eosin; x200 magnification); **(E)** *POLE*-mutant high-grade EC with serous-like features (Hematoxylin and Eosin; x400 magnification); **(F)** p53abn high-grade EC (Hematoxylin and Eosin; x200 magnification); **(G–I)** Depth of invasion ≥50%–Stage IB in *POLE*-mutant EC, NSMP EC, and p53abn EC, respectively (Hematoxylin and Eosin; x5 magnification).

As shown in Figure 5, considering ESGO/ESTRO/ESP 2020 risk classification system based on histopathologic parameters alone, the classes in which integration of molecular classification may result in reallocation are intermediate, high-intermediate, and high risk. One hundred thirty-two (132) of 211 (62.6%) cases would not change risk class regardless of molecular data because either low risk or high risk/advanced. We aimed to propose an algorithm based on histopathologic parameters to select only those cases that could shift the risk class because of the integration of molecular classification and thus changing the post-operative management. As shown in Figure 5 the immunohistochemical profile including the evaluation of the expression of MMR proteins and p53 is essential to characterize all endometrial carcinomas and should be applied to every case. Specifically, evaluation of MMR proteins expression is useful not only for a proper risk group classification but also in selecting patients who possibly need screening for Lynch syndrome, while assessment of p53 may be useful for diagnostic purposes (i.e., histotype) and to identify higher risk cases. Advanced-stage (III and IV) endometrial cancers are at high risk regardless of the molecular subgroup, as well as low risk cases do not need evaluation for *POLE* status. In contrast, all non-low risk patients should be tested for *POLE* mutation. These latter are characterized by having at least one of the following histopathologic features: non-endometrioid histotypes. High-grade, substantial LVSI, stage IB-II (see Figure 6).

According to the proposed algorithm, *POLE* analysis would be appropriate to better define the risk class in 79 (37.4%) patients, whereas it would have been spared in 132 (62.6%) patients, of whom 82 (38.9%) were already at low risk regardless of *POLE* sequencing and 50 (23.7%) had advanced or metastatic disease.

## 4. Discussion

The ESGO/ESTRO/ESP committee has recently proposed a new risk stratification system for endometrial cancer patients incorporating both clinicopathologic and molecular characteristics to overcome the limits of previously adopted classifications (8). As it is known, assignment to the correct risk class has prognostic value and could also affect the appropriate post-operative management of patients and selection of the proper adjuvant treatment. For this reason, misclassification of risk class corresponds to different adjuvant approaches, specifically, it may result in overtreatment or undertreatment. In a previous work by our group, we investigated the prognostic role of additional biomarkers (*ARID1A* and *CTNNB1*) in endometrial carcinoma in a subset of the analyzed cohort showing the relevance of an improved surrogate molecular classification (10). In the present study, expanding the cohort analyzed, we evaluated the impact of the new ESGO/ESTRO/ESP guidelines with the incorporation of molecular subgroups of endometrial carcinoma and we compared them with the previous 2016 recommendations. In our cohort, the recent 2020 risk system resulted in a change of risk class in 32.7% of patients. The reallocation appears to be due to the impact of the molecular data and to a better refinement of histopathologic parameters (i.e., lymphovascular space invasion now defined as focal or substantial). According to the 2020 guidelines, more patients will be allocated to lower risk groups, with 42.2% of patients classified as low and intermediate risk in the 2020 system compared with 25.6% in the 2016 system. Furthermore, taking into account the 2020 risk classification system which includes the difference between “molecular classification unknown” and “known,” the integration of molecular subgroups to the clinicopathologic features allowed the recategorization of 6.6% of patients into a different risk class in our cohort. To the best of our knowledge, our study represents one of the first validations of the ESGO/ESTRO/ESP guidelines in the clinical setting. In agreement with our results, some studies have shown risk group migration in about 6–7% of patients compared with the classification system based on clinicopathologic features alone (29–31). Consistent with our findings, the presence of pathogenic *POLE* mutation or abnormal p53 staining results in a shift to a lower or higher risk class, respectively. This evidence further confirms that surrogate molecular classification of endometrial carcinoma has a prognostic impact and it is, therefore, crucial for appropriate risk class assignment (32).

In addition, we proposed an algorithm based on histopathologic parameters to select only those cases that might need the addition of molecular analysis for appropriate risk classification. Its application to our cohort would result in a reduction of molecular analysis in 132/211 (62.6%) cases without affecting the accuracy of risk class assignment. The algorithm includes immunohistochemical evaluation of MMR proteins and p53 expression in all cases of endometrial carcinoma, whereas *POLE* sequencing is to be restricted to early-stage cases with at least one of the following histopathologic features: (i) non-endometrioid histotypes (i.e., dedifferentiated/undifferentiated carcinoma), (ii) high-grade, (iii) substantial LVSI, (iv) stage IB-II. The reduction of tests would reduce the costs of molecular analysis, thus providing a better allocation of resources. This selection strategy could be useful for the pathologist or multidisciplinary team to easily identify only those cases that need further molecular investigation and can be applied in a resource-limited setting without compromising the accuracy of risk grouping. This algorithm may allow to follow appropriately the current ESGO-ESTRO-ESP guidelines which recommend: (i) avoiding adjuvant treatment for low and intermediate risk patients, including patients with high grade and/or stage II *POLE* mutated endometrial carcinoma; (ii) adding adjuvant brachytherapy or EBRT (external-beam radiation therapy) for high-intermediate risk patients, especially in case with significant LVSI and/or stage II; (iii) reserving EBRT with concurrent adjuvant chemotherapy, or alternatively sequential chemotherapy and radiotherapy, for high-risk patients.

However, it should be considered that our study has a retrospective design belonging to a single institution and lacks a validation cohort to confirm the clinical applicability of our algorithmAs recommended by ISGyP and the ESGO/ESTRO/ESP guidelines, conventional pathologic features such as histotype, grade, myometrial invasion, and lymphovascular space invasion (LVSI) are still important prognostic parameters that allow the majority of cases (60%) to be correctly risk assessed (32, 33). In particular, the prognostic impact of semiquantitative assessment of lymphatic-vascular space invasion has been established in recent studies. In fact, the presence of diffuse LVSI is an independent risk factor for both lymph node metastasis and distant recurrence in endometrial carcinoma patients (34, 35). At the same time, the use of an appropriate immunohistochemical panel to assess MMR and p53 status is crucial for diagnosis, patient management, and risk classification. In particular, MMR deficiency helps to select patients for referral to genetic counseling, while p53 abnormal expression can support the definition of high risk cases. However, it is important to emphasize the importance of adhering to the classification scheme reported by WHO to identify molecular subgroups (13). In fact, considering “multiple-classifier” carcinomas, which in our cohort and other studies are around 7% of cases, evaluation of *POLE* status is necessary to correctly define TCGA surrogate groups (36). Applying the ESGO/ESTRO/ESP guidelines, advanced-stage endometrial carcinomas are defined as high risk regardless of molecular subgroups (including *POLE* tumors) and require adjuvant treatment. For this reason, the proposed algorithm does not take into account molecular analysis in advanced-stage cases. However, recent evidence (36, 37) suggests that molecular classification may guide the appropriate adjuvant treatment in high risk/advanced stage patients. Specifically, adjuvant therapy appears to have different efficacy in each molecular subtype: MMRd tumors may not have the benefit of adding chemotherapy to adjuvant radiotherapy, while p53abn tumors could benefit from adding adjuvant chemotherapy to radiotherapy. These issues regarding adjuvant treatment based on molecular class will be better clarified by the PORTEC-4a trial, TAPER trial (ClinicalTrials.gov: NCT04705649), and TransPORTEC RAINBO program (38, 39). In consideration of the findings now emerging, molecular classification will probably be useful also for predictive purposes in the near future. In addition, as previously reported in our preliminary study, assessment of biomarkers that can help identify patients with the worst prognosis and for whom to reserve more appropriate treatment is critical.

In conclusion, the ESGO/ESTRO/ESP guidelines provide adequate risk stratification and represent a fundamental step in pathologic and molecular integration for a targeted treatment approach. However, in the future it will likely be necessary to consider incorporating additional data for more accurate prognostic stratification of patients, potentially enabling concrete precision medicine. In particular, it might be useful to incorporate additional prognostic markers such as L1CAM, *CTNNB1*, and ARID1A (10, 26, 40–42), and at the same time explore the impact in risk assessment by rare histotypes of aggressive carcinoma (e.g., mesonephric, neuroendocrine, gastric-type carcinomas) currently not included in present guidelines (43).

## Data availability statement

The datasets presented in this study can be found in online repositories. The names of the repository/repositories and accession number(s) can be found below: NCBI Bioproject, PRJNA932605.

## Ethics statement

The studies involving human participants were reviewed and approved by CE-AVEC (Comitato Etico—Area Vasta Emilia Centro, registration n. 27/2019/Sper/AOUBo). The patients/participants provided their written informed consent to participate in this study.

## Author contributions

AL, DB, TM, and CC: conceptualization. AL, TM, DB, and GT: methodology. AL: software. AL, FR, and AC: validation. SC and AL: formal analysis. AL, DB, TM, EG, AA, and MG: investigation. AP, PI, CZ, DR, FA, GD, MT, and SG: resources. AL, DB, AP, DS, VS, DT, AG, and MF: data curation. AL and DB: writing-original draft preparation. CC, GT, DB, PI, and AP: writing-review and editing. AL and MR: visualization. GT, DS, CZ, GR, and GT: supervision. AL: project administration. All authors have read and agreed to the published version of the manuscript.

## Funding

The work reported in this publication was funded by the Italian Ministry of Health, RC-2022-2773478. The research leading to these results has received funding also from AIRC under MFAG 2021 – ID. 26319 – P.I. De Leo Antonio; CARISBO (Cassa di Risparmio di Bologna), grant number 2021.0170 – P.I. de Biase Dario.

## Conflict of interest

DB has received personal fees (as consultant and/or speaker bureau) from Boehringer Ingelheim, and Eli Lilly, unrelated to the current work.

The remaining authors declare that the research was conducted in the absence of any commercial or financial relationships that could be construed as a potential conflict of interest.

## Publisher’s note

All claims expressed in this article are solely those of the authors and do not necessarily represent those of their affiliated organizations, or those of the publisher, the editors and the reviewers. Any product that may be evaluated in this article, or claim that may be made by its manufacturer, is not guaranteed or endorsed by the publisher.

## Footnotes

^1^ http://software.broadinstitute.org/software/igv/

^2^ https://varsome.com/
