# Supplementary material for: Integrated clinicopathologic and molecular analysis of endometrial carcinoma: Prognostic impact of the new ESGO-ESTRO-ESP endometrial cancer risk classification and proposal of histopathologic algorithm for its implementation in clinical practice
Source: Front Med (Lausanne). 2023 Mar 30;10:1146499. doi: 10.3389/fmed.2023.1146499 (PMC10098215; doi:10.3389/fmed.2023.1146499)
Supplement: Supplementary file 1 [file Table_1.DOCX]

Supplementary Material

Integrated clinicopathological and molecular analysis of endometrial carcinoma: prognostic impact of the new ESGO-ESTRO-ESP endometrial cancer risk classification and proposal of histopathological algorithm for its implementation in clinical practice

Dario de Biase^1,2†^, Thais Maloberti^1,3†^, Angelo Gianluca Corradini^4^, Francesca Rosini^4^, Marco Grillini^4^, Martina Ruscelli^3^, Sara Coluccelli^1,3^, Annalisa Altimari^1^, Elisa Gruppioni^1^, Viviana Sanza^1^, Daniela Turchetti^3,5^, Andrea Galuppi^6^, Martina Ferioli^6^, Susanna Giunchi^7^, Giulia Dondi^7^, Marco Tesei^7^, Gloria Ravegnini^2^, Francesca Abbati^8^, Daniela Rubino^8^, Claudio Zamagni^8^, Pierandrea De Iaco^3,7^, Donatella Santini^4^, Claudio Ceccarelli^3^, Anna Myriam Perrone^3,7^, Giovanni Tallini^1,3,4,#^, Antonio De Leo^1,3,4,#,^*

*** Correspondence:** Antonio De Leo: antonio.deleo@unibo.it

# Supplementary Tables

**Supplementary Table 1.** Risk class systems according to ESMO 2016 and ESGO/ESTRO/ESP 2020.

| Risk group | ESMO 2016 | ESGO/ESTRO/ESP 2020  Molecular classification unknown | ESGO/ESTRO/ESP 2020  Molecular classification known |
| --- | --- | --- | --- |
| **Low** | - Stage I endometrioid, grade 1–2, <50% myometrial invasion, LVSI negative | - Stage IA endometrioid + low-grade + LVSI negative or focal | - Stage I–II **POLEmut** endometrial carcinoma, no residual disease  - Stage IA **MMRd/NSMP** endometrioid carcinoma + low-grade + LVSI negative or focal |
| **Intermediate** | - Stage I endometrioid, grade 1–2, ≥50% myometrial invasion, LVSI negative | - Stage IB endometrioid + low-grade + LVSI negative or focal  - Stage IA endometrioid + high-grade + LVSI negative or focal  - Stage IA non-endometrioid (serous, clear cell, undifferentiared carcinoma, carcinosarcoma, mixed) without myometrial invasion | - Stage IB **MMRd/NSMP** endometrioid carcinoma + low-grade + LVSI negative or focal  - Stage IA **MMRd/NSMP** endometrioid carcinoma + high-grade + LVSI negative or focal  - Stage IA **p53abn** and/or non-endometrioid (serous, clear cell, undifferentiated carcinoma, carcinosarcoma, mixed) without myometrial invasion |
| **High–intermediate** | - Stage I endometrioid, grade 3, <50% myometrial invasion, regardless of LVSI status  Stage I endometrioid, grade 1–2, LVSI unequivocally positive, regardless of depth of invasion | - Stage I endometrioid + substantial LVSI regardless of grade and depth of invasion  - Stage IB endometrioid high-grade regardless of LVSI status  - Stage II | - Stage I **MMRd/NSMP** endometrioid carcinoma + substantial LVSI regardless of grade and depth of invasion  - Stage IB **MMRd/NSMP** endometrioid carcinoma high-grade regardless of LVSI status  - Stage II **MMRd/NSMP** endometrioid carcinoma |
| **High** | -Stage I endometrioid, grade 3, ≥50% myometrial invasion, regardless of LVSI status  - Stage II  - Stage III endometrioid, no residual disease  - Non-endometrioid (serous or clear-cell or undifferentiated carcinoma, or carcinosarcoma) | - Stage III–IVA with no residual disease  - Stage I–IVA non-endometrioid (serous, clear cell, undifferentiated carcinoma, carcinosarcoma, mixed) with myometrial invasion, and with no residual disease | - Stage III–IVA **MMRd/NSMP** endometrioid carcinoma with no residual disease  - Stage I–IVA **p53abn** endometrial carcinoma with myometrial invasion, with no residual disease  - Stage I–IVA **NSMP/MMRd** serous, undifferentiated carcinoma, carcinosarcoma with myometrial invasion, with no residual disease |
| **Advanced/ metastatic** | - Stage III residual disease and stage IVA  - Stage IVB | - Stage III–IVA with residual disease  - Stage IVB | - Stage III–IVA with residual disease of any molecular type  - Stage IVB of any molecular type |

| **Supplementary Table 2**: List of antibodies | | | |
| --- | --- | --- | --- |
| Antibody | Source | Dilution | Method |
| Rabbit anti-PTEN, clone SP218 | Ventana, USA | RTU | UltraCC1 x 56' at 100°C - Ab 16' at 36°C |
| Mouse anti-p53, clone DO7 | Ventana, USA | RTU | UltraCC1 x 24' at 95°C - Ab 12' at 36°C |
| Mouse anti-MLH1, clone M1 | Ventana, USA | RTU | UltraCC1 x 56' at 98°C - Ab 32' at 36°C |
| Mouse anti-PMS2, clone A16-4 | Ventana, USA | RTU | UltraCC1 x 64' at 99°C - Ab 32' at 36°C |
| Mouse anti-MSH2, clone G219-1129 | Ventana, USA | RTU | UltraCC1 x 56' at 95°C - Ab 32' at 36°C |
| Rabbit anti-MSH6, clone SP93 | Ventana, USA | RTU | UltraCC1 x 64' at 100°C - Ab 12' at 36°C |
| Rabbit anti-Ki67, clone 30-9 | Ventana, USA | RTU | UltraCC1 x 32' at 99°C - Ab 8' at RT |
| Visualization with OptiView DAB Detection kit, Ventana, USA | | |  |
